# Design Features of Randomized Clinical Trials of Vitamin D and Falls: A Systematic Review

**DOI:** 10.3390/nu10080964

**Published:** 2018-07-26

**Authors:** Olive Tang, Stephen P. Juraschek, Lawrence J. Appel

**Affiliations:** 1 Johns Hopkins School of Medicine, Baltimore, MD 21287, USA; otang@jhmi.edu (O.T.); sjurasch@bidmc.harvard.edu (S.P.J.); 2 Johns Hopkins Bloomberg School of Public Health, Baltimore, MD 21287, USA; 3 The Welch Center for Prevention, Epidemiology and Clinical Research, Johns Hopkins University, Baltimore, MD 21287, USA; 4 Beth Israel Deaconess Medical Center, Boston, MA 02215, USA; 5 Harvard Medical School, Boston, MA 02115, USA

**Keywords:** vitamin D, falls prevention, trials

## Abstract

Recent guidelines have advocated against the use of vitamin D supplementation as a means to prevent falls in older adults. However, meta-analyses of the available trials have reached divergent conclusions, and the key design features of these trials have not been well characterized. We conducted a systematic review of 30 randomized trials that reported the effects of vitamin D supplements on falls. Trials were identified by reviewing references of published meta-analyses and updated with a systematic PubMed search. We assessed three key design features: (1) recruitment of participants with vitamin D deficiency or insufficiency; (2) provision of daily oral vitamin D supplementation; and (3) utilization of highly sensitive at-event falls ascertainment. The trials enrolled a median of 337 (IQR: 170-1864) participants. Four (13.3%) trials restricted enrollment to those who were at least vitamin D insufficient, 18 (60.0%) included at least one arm providing daily supplementation, and 16 (53.3%) used at-event reporting. There was substantial heterogeneity between trials, and no single trial incorporated all three key design features. Rather than concluding that vitamin D is ineffective as a means to prevent falls, these findings suggest that existing trial evidence is insufficient to guide recommendations on the use of vitamin D supplements to prevent falls.

## 1. Introduction

One in four Americans over the age of 65 fall each year, with one requiring emergency care every 11 seconds and one resulting in death every 19 minutes [1]. In addition to the substantial morbidity and mortality from falls, associated medical costs are substantial, with over 31.9 billion dollars spent on direct falls-related healthcare in 2015 [2]. The prevalence of falls and their related costs continue to rise, highlighting the need for improved falls prevention programs. 

Vitamin D deficiency is common in older adults [3] and has been associated with falls [4,5], muscle loss [6] and frailty [7,8]. Early trials of vitamin D supplementation in preventing falls demonstrated a reduction in risk compared to non-supplemented controls [9,10,11]. Based on this, the United States Preventative Services Task Force (USPSTF) initially adopted vitamin D supplementation among their recommendations for falls prevention in older adults [12]. However, more recent trials have challenged this association, with some trials showing no effect [13,14,15] or in some cases, an increased risk of falls [16,17]. Subsequently, recent updates from the USPSTF have begun recommending against the use of vitamin D in falls prevention in older adults without vitamin D deficiency [18]. Whether there are benefits of vitamin D supplementation in the prevention of falls remain disputed. 

The reasons for these conflicting results continue to be unclear despite multiple systematic reviews and meta-analyses. Notably, each of the existing reviews do not include the same studies in their pooled estimates due to differences in pre-specified inclusion criteria. The subsequent pooled estimates conflict and heterogeneity between trials, particularly in their design features, continues to be under-characterized [9,10,11,19,20,21]. Greater understanding of variabilities in trial design could be critical to inform the next generation of trials to better guide recommendations of vitamin D supplementation in falls prevention.

In this paper, we systematically reviewed how trials incorporated vitamin D levels in their inclusion criterion, administered the vitamin D intervention, and ascertained falls as the outcome of interest. We focused on three aspects that may be important based on the following principles of trial design. The first is enrichment of the study population, which refers to the recruitment of participants who are likely to benefit from the intervention and in whom this benefit is most likely to be detectable [22,23,24]. In the case of vitamin D trials, this consists of those who are vitamin D insufficient or deficient [24], defined as serum 25-hydroxyvitamin D (25(OH)D) levels below 30 ng/mL, according to clinical guidelines from The Endocrine Society [25], which have been used to monitor prevalence of vitamin D insufficiency in the U.S. [26].

The second characteristic is the administration of the intervention, as there are suspected physiological differences between daily oral dosing and bolus dosing of vitamin D. There has been recent interest in the use of large bolus dosing of vitamin D supplementation, in large part to improve compliance as compared to daily administration [27]. However, emerging work suggests that this approach may be associated with increased risks of harms, including falls and fractures, particularly in older adults [28]. While our understanding of the impacts of bolus dosing is incomplete, daily oral administration more closely reflects natural dietary exposure to vitamin D and thus may remain the regimen that maximizes potential benefits while minimizing potential risks to participants.

Finally, sensitive outcome ascertainment methods are important in trials in order to reduce recall bias and to improve the power of trials to detect possible differences between treatment arms. A direct comparison of falls ascertainment by falls calendar compared to recall over 3 months in the Maintenance of Balance, Independent Living, Intellect, and Zest in the Elderly Boston Study, found that recall at 3 months missed a quarter of the participants who fell [29]. In older adults, in particular, recall bias on falls questionnaires has been well documented, especially when complicated by possible cognitive decline [30]. A consensus statement from the Prevention of Falls Network Europe advocated the importance of daily event recording in the ascertainment of falls in trials [31], further highlighting that at-event reporting such as through falls diaries, calendars, or nursing report is crucial in fall prevention trials. 

In this context, we performed a systematic review of trials that reported the effects of vitamin D supplementation on falls with a focus on the key design features outlined above. 

## 2. Materials and Methods

*Trial search and selection*. To identify relevant trials, we searched the references of prior published meta-analyses, the latest of which included trials published before 31 December 2013 [9,10,11,19,20,21], and updated this list using a PubMed search for “Randomized Controlled Trial”, “vitamin D” and “Falls” for trials published between 1 January 2014 and 1 October 2017. Our list was further supplemented by bibliography review of the identified trials. Inclusion criteria were: (1) randomized controlled trial (RCT); (2) vitamin D supplementation in any form, with or without concomitant calcium; and (3) falls reported as a primary or secondary outcome. We excluded: (1) trials in pregnant women or children; (2) cluster-randomized trials; (3) trials assessing vitamin D supplementation as part of a multifactorial program; and (4) trials assessing falls only as part of adverse event reporting. To analyze the design features of ongoing trials, we queried clinicaltrials.gov for those with a status of “not yet recruiting”, “recruiting”, or “active” with “vitamin D” as an intervention and “falls” as an outcome. Seventeen entries matched these criteria. We excluded non-randomized studies (*n* = 4), those including vitamin D as a part of the standard care comparison (*n* = 1) or broader prevention program (*n* = 5), those assessing only falls that lead to fractures (*n* = 1) or epileptic falls (*n* = 1). Five ongoing randomized control trials remained. 

*Data ascertainment*. We extracted trial characteristics including distributions of age, sex, trial size, supplementation regimen, and outcome ascertainment methods from trial publications. Our findings were synthesized as percentages for binary variables or mean/median (standard deviation/interquartile range) for continuous variables. Authors were not contacted for additional information. Analyses were conducted using Microsoft Excel v15.40. 

*Key trial features*. We approached this review with the a priori hypothesis that important trial characteristics in assessing vitamin D supplementation in falls prevention include: (1) enrolling participants in need of the intervention, defined as those with vitamin D deficiency or insufficiency (baseline serum 25(OH)D <30 ng/mL); (2) administering daily oral vitamin D supplementation; and (3) using highly sensitive at-event ascertainment mechanisms that minimize recall bias, such as falls diary, falls calendar, mailed postcard at time of fall, or nursing report. 

## 3. Results

We identified 30 RCTs, conducted in 11 countries over 2 decades, which reported on the effect of vitamin D supplementation on falls (Supplemental Table S1). Twenty-six of these were non-factorial, parallel-arm trials, and 4 were factorial. Intervention duration ranged from 8 weeks to 5 years.

### 3.1. Participant Recruitment and Baseline

Trials included between 96 and 9440 participants (median: 337, IQR = 170–1864). Of these trials, 83.3% included participants with baseline mean or median age greater than 70, and 40.0% recruited only individuals over 70 (Table 1). Most trials included predominantly women (average 80.3%), with 43.3% including only women (Table 1). Nine trials (30.0%) explicitly recruited adults with a history of prior falls (Supplemental Table S2).

### 3.2. Vitamin D Insufficiency

Almost half (46.7%) of the trials did not exclude participants who were already taking ≥200 IU/day of vitamin D supplements prior to enrollment (Table 1). All but 1 examined 25(OH)D levels either at baseline, follow-up, or both (Supplemental Table S3). Only 3 trials exclusively recruited vitamin D-deficient participants, and 1 additional trial recruited those who were vitamin D insufficient. Three of the 4 trials found at least one dose of vitamin D supplementation was associated with a reduction in falls, while the fourth reported a non-significant reduction (Table 2). Among the 25 trials reporting a mean or median baseline 25(OH)D level, participants in 60% of the trials were on average vitamin D deficient, while 96.0% of trials included those who were, on average, at least vitamin D insufficient. Laboratory methods to estimate 25(OH)D levels varied widely, with radioimmunoassay (RIA) being the most popular (48.3%) (Table 1). Four trials (13.8%) utilized liquid chromatography followed by tandem mass spectrometry (Table 1).

### 3.3. Vitamin D Intervention

Vitamin D supplementation approaches were variable, with 7 trials exploring multiple treatment arms. Across the 30 trials, there were 46 treatment arms: 67.4% provided daily oral supplements, 8.7% provided high-dose intramuscular injections (Table 1) and 8.7% provided yearly supplementation (Supplemental Table S4). At least one bolus-dosing regimen was included in 43.3% of trials, 10 of which used oral bolus dosing and 3 intramuscular injections (Supplemental Table S4). All three trials reporting an increased risk of falls with supplementation used bolus dosing (Table 2). Over a quarter of the treatment arms provided concomitant calcium (Table 1). Compliance was reported by 21 (70.0%) trials (Supplemental Table S4).

### 3.4. Falls Assessment

Over half (53.3%) of the trials assessed falls as a primary outcome, and 16 trials used prospective at-event reporting methods (falls diary, calendar, postcard or nursing report) as their principal outcome ascertainment method. Less than half (43.3%) relied exclusively on post hoc verbal recall through questionnaires or interviews (Table 1). All trials reported number of participants who fell, but only 40.0% reported recurrent falls (Supplemental Table S5).

### 3.5. Key Characteristics

No single trial incorporated all three design characteristics of interest. However, 11 of the 30 trials included 2 of the 3 features: 8 provided daily oral supplementation and at-event capturing, but did not exclusively recruit vitamin D-insufficient participants; 2 recruited vitamin D-insufficient participants and provided daily supplementation; and 1 recruited vitamin D-insufficient participants and utilized at-event falls ascertainment.

## 4. Discussion

Conflicting results from trials reporting on the impact of vitamin D supplements on falls and divergent conclusions from subsequent meta-analyses have made it difficult to reach a consensus on the role of vitamin D supplements as a means to prevent falls. Our review of design features related to inclusion criteria, intervention and outcome ascertainment showed that no single trial possessed all three key characteristics of recruiting an at-risk population with insufficient or deficient vitamin D levels, providing daily supplementation, and using an at-event falls reporting method. These findings demonstrate substantial methodologic heterogeneity among existing trials, potentially contributing to why meta-analyses have been inconclusive with regards to the use of vitamin D supplements as a means to prevent falls. Hence, additional trials that incorporate these features are still needed.

Available systematic reviews and meta-analyses have reached conflicting conclusions regarding the efficacy of vitamin D supplementation in falls prevention. Some have concluded a protective effect of supplementation [9,11,20] while others have failed to find any effect [19,32,33,34]. Each meta-analysis incorporated different subsets of trials, pooling between 9 [9] and 54 trials [21], based on differences in inclusion criteria. For example, some exclude vitamin D analogues or metabolites (*n* = 26 trials) [19], while others assessed only trials incorporating intermittent high-dose vitamin D supplementation (*n* = 9 trials) [32]. As a result, despite the large volume of research on vitamin D in falls prevention, definitive evidence remains elusive.

In our study, we focused on the presence of three key characteristics, which were identified a priori to likely impact findings in trials that test the effect of vitamin D supplements on falls. The first was study population enrichment, namely, the recruitment of vitamin D-insufficient or -deficient participants (baseline 25(OH)D <30 ng/mL), an at-risk population, who would most likely benefit from intervention. Most trials observed a mean or median baseline 25(OH)D <30 ng/mL, with 4 trials having this as an established inclusion criteria. Of these 4 trials, 3 recruited exclusively those who were vitamin D deficient (25(OH)D <20 ng/mL). Trials not focusing on this at-risk population may be more likely to observe null results as prior work suggests vitamin D supplementation may only reduce falls in those with low baseline vitamin D [35]. The importance of this feature has also been highlighted by Heaney based on analyses of nutrient response curves [24].

The second key characteristic was vitamin D supplementation via oral, daily dosing compared to bolus dosing, either orally or via intramuscular injection. Oral daily dosing more closely mimics usual dietary intake of vitamin D; in contrast, bolus dosing of vitamin D is controversial. In particular, high dose intramuscular injection preparations have been found to have high variability and are not currently approved by the U.S. Food and Drug Administration [36]. While the use of non-physiologic dosing may be associated with an increase in the risk of falls [32,37], some have nonetheless advocated for this approach as it improves compliance among participants [28]. In our sensitivity analysis allowing for bolus dosing approaches, a single parallel-arm trial, analyzing the 6-month follow-up after a single intramuscular injection of 600,000 IU vitamin D2, recruited 139 participants with baseline 25(OH)D ≤12 ug/L and used falls diaries to capture events. Additional work is needed to better understand the tradeoff between possible risks and the benefits of compliance associated with bolus administration.

The third key characteristic in our review was the use of sensitive at-event ascertainment methods, such as a falls diary, calendar, mailed postcard at the time of fall, or nurse reporting. These methods of fall ascertainment minimize recall bias. Prior reviews of recall bias in community-dwelling older adults highlighted these concerns of missing events, especially with non-injurious falls, based on interval recall, a problem further exacerbated in those with cognitive decline [30]. Thus, recall-based methods may be more prone to bias results towards the null, especially in older populations when assessing frequency of falls or time to first fall.

No single trial was designed with all 3 of the features assessed. Although over a third incorporated 2 of the 3 characteristics, these subsets remained heterogeneous in other elements such as overall design (factorial vs. parallel-arm), size, duration, use of concomitant calcium, and reported outcome (recurrent fallers, time to first fall, etc.). This residual diversity in design choices further complicate efforts to synthesize across trials.

Among the 5 ongoing randomized trials of vitamin D supplementation registered on clinicaltrials.gov (as of 21 June 2018) with falls as a primary or secondary outcome, 2 trials are focused on the general older adult population: Vitamin D and Muscle Function Study (EVIDENCE) and Study To Understand Fall Reduction and Vitamin D in You (STURDY). Both have incorporated all three key design features. Future guidelines for the use of vitamin D in the prevention of falls in the general older adult population may benefit from the findings of these 2 ongoing trials and future trials of similar design. The other 3 trials, focused on vitamin D in preventing side effects of androgen depravation therapy, cardiovascular disease, and falls in pre-frail hypogonadal men did not incorporate all 3 features: only 1 recruited vitamin D-insufficient participants, only 1 indicated at-event outcomes reporting (2 did not specify their falls assessment method), and all 3 used bolus dosing. The design of these 5 ongoing trials highlight a growing, though by no means universal, appreciation of the importance of these design features.

Our review has limitations. First, our three key design features might be viewed as incomplete. There are additional considerations such as recruitment of participants with a history of falls, provision of adequate doses of supplementation, or concomitant use of calcium, which may be important to consider. However, the addition of other criteria would not change our conclusion that no trial has all three features we have highlighted. Second, while our review was systematic and comprehensive, there is always a possibility that smaller studies or pilot trials were not captured by our review approach. Further, we excluded trials that assessed falls as part of adverse event reporting [38,39,40]. However, as none of these trials collected outcomes data through prospective at-event reporting, this exclusion would not impact our overall conclusion. Third, we did not contact the authors to obtain additional information about trial methodologies. Finally, we made an a priori decision not to conduct a meta-analysis given the profound design heterogeneity between trials and the absence of trials that met our three key design criteria. This decision should be revisited with the publication of future well-designed trials.

A significant strength of our review is our decision to include all trials studying vitamin D supplementation with reported falls data, regardless of design or form of supplementation. This approach allowed us to better capture the heterogeneity of the trials to date and to identify ongoing gaps where further work is needed.

Our analysis has policy implications. First, the absence of trials incorporating all three features in their design demonstrates the ongoing need for trials assessing the effects of vitamin D supplements on falls. In 2013, the National Institute on Aging (NIA) released a RFA (RFA-AG-14-001) requesting an adequately powered trial that focused on at-risk populations of older community-dwelling adults. Second, recommendations on vitamin D as a means to prevent falls should not be guided by existing meta-analyses or updated meta-analyses given the methodological limitations of available trials. Previously, Bolland et al. (2014) concluded that simply pooling additional data from more trials will not likely alter the current conclusions of their meta-analysis [19]. We believe that a potential reason for their observed null effect might be design limitations of prior trials rather than a lack of efficacy.

In conclusion, despite numerous trials and subsequent meta-analyses performed, the efficacy of vitamin D supplementation as a means to prevent falls remains uncertain. Available trials vary substantially with none possessing all three key features related to study population, intervention administration, and outcome ascertainment. Given the inconclusive evidence and substantial heterogeneity in trial designs, rather than suggest vitamin D is ineffective, our findings demonstrate the importance of further trials on vitamin D and falls, and highlight 3 key characteristics these trials should address: vitamin D deficiency, vitamin D administration, and at-event falls ascertainment.

## Figures and Tables

**Table 1:** Summary of Trial Characteristics.

Participant Characteristics	Frequency, *n*/30 (%)
Age	
Mean or Median ≥70	25 (83.3)
All participants ≥70 *	12 (40.0)
% women	
Median, % (IQR)	80.3 (61–100)
100% women *	13 (43.3)
Trial size, median (IQR)	337 (170–1864)
Recruitment with falls history *	9 (30.0)
Non-exclusion of individuals taking ≥200 IU *^,†^	14 (46.7)
Vitamin D Measurement and Intervention	
Laboratory Assessment of vitamin D Levels	
Not assessed at any time point, *n* (%)	1 (3.3)
Assessed at follow-up, *n* (%)	28 (93.3)
Assay Type, *n*/29 ^‡^ (%)	
Radioimmunoassay	14 (48.3)
Protein binding assay	2 (6.9)
High-performance liquid chromatography	2 (6.9)
Immunoenzymometric assay	1 (3.4)
Liquid chromatography with tandem mass spectrometry	4 (13.8)
Enzyme linked immunoassay	1 (3.4)
Chemiluminescent immunoassay	1 (3.4)
Not reported	3 (10.3)
Baseline 25(OH)D	
Baseline mean or median <30 ng/mL, n/25 ^§^ (%)	24 (96.0)
<20 ng/mL, *n*/25 ^§^ (%)	15 (60.0)
All participants with baseline <30 ng/mL *, *n*/30 (%)	4 (13.3)
<20 ng/mL *, *n*/30 (%)	3 (10.0)
Intervention, *n*/46 treatment arms (%)	
Daily, oral	31 (67.4)
Non-daily, oral	11 (23.9)
Intramuscular	4 (8.7)
Concomitant calcium	12 (26.1)
Principal Outcomes Assessment Method	
Falls as primary outcome	17 (56.7)
Event Ascertainment	
At-event reporting	16 (53.3)
Falls Diary	8 (26.7)
Postcard/Calendar	3 (10.0)
Nurse report	5 (16.7)
Interview/Questionnaire	14 (43.3)
Nursing Home Chart Review	1 (3.3)

* trial enrollment criteria; ^†^ explicit dose exclusion was not reported; multivitamin use was permitted (Broe et al. 2009); ^‡^ among trials that measured 25(OH)D either at baseline and/or follow-up; ^§^ trials were excluded from the denominator if they did not assess baseline 25(OH)D (3 trials) or the median/mean baseline level was not clear from the primary publication (2 trials).

**Table 2:** Trial findings by trial characteristic.

	Beneficial	Null Effect	Harmful
Overall	13 *^,†,‡^	14	3
Baseline vitamin D			
All deficient	3 *	1	0
Mean/Median deficient	11 *^,†,‡^	10	3
Vitamin D supplementation			
Daily Oral	11 *^,†^	7	0
Outcome ascertainment			
At-event reporting	7 ^‡^	7	2

* trial reporting a U-shaped relationship by vitamin D level; ^†^ trial reporting a significant effect by Poisson but not negative binomial distribution; ^‡^ trial reporting significant results after adjustment for baseline characteristics.

## Supplementary Materials

The following are available online at http://www.mdpi.com/2072-6643/10/8/964/s1, Table S1: List of included vitamin D and Falls Trials, Table S2: Trial recruitment and methodology characteristics, Table S3: vitamin D assessment at baseline and follow-up, Table S4: Trial intervention and follow-up characteristics, Table S5: Outcome assessment and reporting characteristics.
