# Supplementary material for: Design Features of Randomized Clinical Trials of Vitamin D and Falls: A Systematic Review
Source: Nutrients. 2018 Jul 26;10(8):964. doi: 10.3390/nu10080964 (PMC6115709; doi:10.3390/nu10080964)
Supplement: Supplementary file 1 [file nutrients-10-00964-s001.pdf]

APPENDIX

Supplemental Table S1: List of included Vitamin D and Falls Trials

	Authors	Publication year	Recruitment start year	Country	Citation
1	Graafmans et al.	1996	1992	Netherlands	Graafmans WC, Ooms ME, Hofstee HMA, Bezemer PD, Bouter LM, Lips P. Falls in the Elderly: A Prospective Study of Risk Factors and Risk Profiles. Am J Epidemiol. 1996;143(11):1129-1136. doi:10.1093/oxfordjournals.aje.a008690.
2	Pfeifer et al.	2000	1997	Germany	Pfeifer M, Begerow B, Minne HW, et al. Effects of a short-term vitamin D and calcium supplementation on body sway and secondary hyperparathyroidism in elderly women. J Bone Min Res. 2000;15(6):1113-1118. doi:10.1359/jbmr.2000.15.6.1113.
3	Bischoff et al.	2003	1999	Switzerland	Bischoff HA, Stahlein HB, Dick W, et al. Effects of Vitamin D and Calcium Supplementation on Falls: J Bone Miner Res. 2003;18(2):343-351.
4	Latham et al.	2003	1999	New Zealand/ Australia	Latham N, Anderson CS, Lee A, Bennet DA, Moseley A, Cameron ID. A Randomized, Controlled Trial of Quadriceps Resistance Exercise and Vitamin D in Frail Older People: The Interventions Trial in Elderly Subjects (FITNESS). 2003:291-299.
5	Trivedi et al.	2003	1996	UK	Trivedi DP, Doll R, Khaw KT. Effect of four monthly oral vitamin D3 (cholecalciferol) supplementation on fractures and mortality in men and women living in the community: randomised double blind controlled trial. BMJ. 2003;326(7387):469. doi:10.1136/bmj.326.7387.469.
6	Dhesi et al.	2004	1999	UK	Dhesi JK, Jackson SHD, Bearne LM, et al. Vitamin D supplementation improves neuromuscular function in older people who fall. Age Ageing. 2004;33(6):589-595. doi:10.1093/ageing/afh209.
7	Harwood et al.	2004	NR	UK	Harwood RH, Sahota O, Gaynor K, Masud T, Hosking DJ. A randomised, controlled comparison of different calcium and vitamin D supplementation regimens in elderly women after hip fracture: The Nottingham Neck of Femur (NoNOF) study. Age Ageing. 2004;33(1):45-51. doi:10.1093/ageing/afh002.
8	Flicker et al.	2005	1996	Australia	Flicker L, MacInnis RJ, Stein MS, et al. Should older people in residential care receive vitamin D to prevent falls? Results of a randomized trial. J Am Geriatr Soc. 2005;53(11):1881-1888. doi:10.1111/j.1532-5415.2005.00468.x.
9	Grant et al.	2005	1999	UK	The RECORD Trial Group. Oral vitamin D3 and calcium for secondary prevention of low-trauma fractures in elderly people (Randomised Evaluation of Calcium or vitamin D, RECORD): A randomised placebo-controlled trial. Lancet. 2005;365(9471):1621-1628. doi:10.1016/S0140-6736(05)63013-9.

10	Porthouse et al.	2005	1999	UK	Porthouse J, Cockayne S, King C, et al. Randomised controlled trial of calcium and supplementation with cholecalciferol (vitamin D3) for prevention of fractures in primary care. BMJ. 2005;330(7498):1003. doi:10.1136/bmj.330.7498.1003.
11	Bischoff-Ferrari et al.	2006	NR	USA	Bischoff-Ferrari HA, Orav EJ, Dawson-Hughes B. Effect of cholecalciferol plus calcium on falling in ambulatory older men and women: a 3-year randomized controlled trial. Arch Intern Med. 2006;166(4):424-430. doi:10.1016/S0084-3741(08)70160-4
12	Broe et al.	2007	1995	USA	Broe KE, Chen TC, Weinberg J, Bischoff-Ferrari HA, Holick MF, Kiel DP. A higher dose of vitamin D reduces the risk of falls in nursing home residents: A randomized, multiple-dose study. J Am Geriatr Soc. 2007;55(2):234-239. doi:10.1111/j.1532-5415.2007.01048.x.
13	Burleigh et al.	2007	NR	UK	Burleigh E, McColl J, Potter J. Does vitamin D stop inpatients falling? A randomised controlled trial. Age Ageing. 2007;36(5):507-513. doi:10.1093/ageing/afm087.
14	Smith et al.	2007	1998	UK	Smith H, Anderson F, Raphael H, Maslin P, Crozier S, Cooper C. Effect of annual intramuscular vitamin D on fracture risk in elderly men and women - A population-based, randomized, double-blind, placebo-controlled trial. Rheumatology. 2007;46(12):1852-1857. doi:10.1093/rheumatology/kem240.
15	Prince et al.	2008	2003	Australia	Prince RL, Austin N, Devine A, Dick IM, Bruce D, Zhu K. Effects of Ergocalciferol Added to Calcium on the Risk of Falls in Elderly High-Risk Women. Arch Intern Med. 2008;168(1):103. doi:10.1001/archinternmed.2007.31.
16	Pfeifer et al.	2009	2001	Germany/ Austria	Pfeifer M, Begerow B, Minne HW, Suppan K, Fahrleitner-Pammer A, Dobnig H. Effects of a long-term vitamin D and calcium supplementation on falls and parameters of muscle function in community-dwelling older individuals. Osteoporos Int. 2009;20(2):315-322. doi:10.1007/s00198-008-0662-7.
17	Kärkkäinen et al.	2010	2003	Finland	Kärkkäinen MK, Tuppurainen M, Salovaara K, et al. Does daily vitamin D 800 IU and calcium 1000 mg supplementation decrease the risk of falling in ambulatory women aged 65-71 years? A 3-year randomized population-based trial (OSTPRE-FPS). Maturitas. 2010;65(4):359-365. doi:10.1016/j.maturitas.2009.12.018.
18	Sanders et al.	2010	2003	Australia	Sanders KM, Stuart AL, Williamson EJ, Simpson JA, Kotowicz MA, Young D et al. Annual high-dose oral vitamin D and falls and fractures in older women. Jama. 2010;303(18):1815-1822.
19	Glendenning et al.	2012	2009	Australia	Glendenning P, Zhu K, Inderjeeth C, Howat P, Lewis JR, Prince RL. Effects of three-monthly oral 150,000 IU cholecalciferol supplementation on falls, mobility, and muscle strength in older postmenopausal women: A randomized controlled trial. J Bone Miner Res. 2012;27(1):170-176. doi:10.1002/jbmr.524.

20	Law et al.	2006	NR	UK	Law M, Withers H, Morris J, Anderson F. Vitamin D supplementation and the prevention of fractures and falls: Results of a randomised trial in elderly people in residential accommodation. Age Ageing. 2006;35(5):482-486. doi:10.1093/ageing/afj080.
21	Bischoff-Ferrari et al.	2016	2009	Switzerland	Bischoff-Ferrari HA, Dawson-Hughes B, Orav EJ, et al. Monthly High-Dose Vitamin D Treatment for the Prevention of Functional Decline. JAMA Intern Med. 2016;176(2):175. doi:10.1001/jamainternmed.2015.7148
22	Uusi-Rasi et al.	2015	2010	Finland	Uusi-Rasi K, Patil R, Karinkanta S, et al. Exercise and Vitamin D in Fall Prevention Among Older Women. JAMA Intern Med. 2015;175(5):703. doi:10.1001/jamainternmed.2015.0225.
23	Dukas et al.	2004	2000	Switzerland	Dukas L, Bischoff H, Lindpaintner LS, et al. Alfacalcidol Reduces the Number of Fallers in a Community- Dwelling Elderly Population with a Minimum Calcium Intake of More Than 500 Mg Daily. Endocr Rev. 2004:230-236.
24	Gallagher et al.	2001	NR	USA	Gallagher JC, Fowler SE, Detter JR, Sherman SS. Combination treatment with estrogen and calcitriol in the prevention of age-related bone loss. J Clin Endocrinol Metab. 2001;86(8):3618-3628. doi:10.1210/jc.86.8.3618.
25	Cangussu et al.	2015	2013	Brazil	Cangussu LM, Nahas-Neto J, Orsatti CL, et al. Effect of isolated vitamin D supplementation on the rate of falls and postural balance in postmenopausal women fallers. Menopause. 2015;23(3):1. doi:10.1097/GME.0000000000000525.
26	Wood et al.	2014	2009	UK	Wood AD, Secombes KR, Thies F, et al. A parallel group double-blind RCT of vitamin D3 assessing physical function: Is the biochemical response to treatment affected by overweight and obesity? Osteoporos Int. 2014;25(1):305-315. doi:10.1007/s00198-013-2473-8.
27	Ginde et al.	2017	2010	USA	Ginde AA, Blatchford P, Breese K, et al. High-Dose Monthly Vitamin D for Prevention of Acute Respiratory Infection in Older Long-Term Care Residents: A Randomized Clinical Trial. J Am Geriatr Soc. 2017;65(3):496-503. doi:10.1111/jgs.14679.
28	Houston et al.	2015	2010	USA	Houston DK, Tooze JA, Demons JL, et al. Delivery of a Vitamin D intervention in homebound older adults using a meals-on-wheels program: A pilot study. J Am Geriatr Soc. 2015;63(9):1861-1867. doi:10.1111/jgs.13610.
29	Smith et al.	2017	2008	USA	Smith LM, Gallagher JC, Suiter C. Medium doses of daily vitamin D decrease falls and higher doses of daily vitamin D3 increase falls: A randomized clinical trial. J Steroid Biochem Mol Biol. 2017;173(March):317-322. doi:10.1016/j.jsbmb.2017.03.015.

30	Khaw et al.	2017	2011	New Zealand	Khaw KT, Stewart AW, Waayer D, et al. Effect of monthly high-dose vitamin D supplementation on falls and non-vertebral fractures: secondary and post-hoc outcomes from the randomised, double-blind, placebo-controlled ViDA trial. Lancet Diabetes Endocrinol. 2017;5(6):438-447. doi:10.1016/S2213-8587(17)30103-1.

Supplemental Table S2: Trial recruitment and methodology characteristics

Trial	N enrolled	Trial Design	% Women	Age		Exclusion of VitD supplementation	Facilitated Living/ Care Facility	Prior falls/fracture as eligibility criteria	Description of randomization process	Blinding	
				Eligibility Criteria	Mean or [Median]					Participant	Outcome Assessor
1	368	Parallel	85.0	70+	51% >83	None	Y	Y	NR	Y	NR
2	148	Parallel	100.0	70+	74.0	All	N	N	NR	Y	Y
3	122	Parallel	100.0	60+	85.3	None	Y	N	Y	Y	Y
4	243	Factorial	53.0	65+	79.1	None	Y	N	Y	Y	Y
5	2686	Parallel	24.2	65-85	74.8	All	N	N	Y	Y	Y
6	139	Parallel	77.7	65+	76.8	All	N	Y	Y	Y	Y
7	150	Parallel	100.0	NC	81.2	None	Y	Y	Y	Y	N
8	625	Parallel	94.9	NC	83.5	All	Y	N	Y	Y	Y
9	5292	Factorial	85.0	70+	77.0	≥200IU	N	Y	Y	Y	Y
10	3314	Parallel	100.0	70+	76.8	None	N	N	Y	NR	NR
11	445	Parallel	55.3	65+	70.8	None	N	N	Y	Y	Y
12	124	Parallel	73.0	NC	89.0	None	Y	N	Y	Y	Y
13	205	Parallel	59.0	65+	[84]	All	Y	N	Y	Y	Y
14	9440	Parallel	53.9	75+	[79.1]	≥400IU	N	N	Y	Y	Y
15	302	Parallel	100.0	70-90	77.2	All	N	Y	Y	Y	Y
16	242	Parallel	74.5	70+	76.5	All	N	N	NR	Y	Y
17	3139	Parallel	100.0	65+	67.4	None	N	N	Y	N	N
18	2256	Parallel	100.0	70+	[76]	≥400IU	N	N	Y	Y	Y
19	686	Parallel	100.0	70+	76.7	All	N	N	Y	Y	Y
20	3717	Parallel	76.0	60+	85.0	All	Y	N	Y	N	Y
21	200	Parallel	67.0	70+	78.0	≥800IU	N	Y	Y	Y	Y
22	409	Factorial	100.0	70-80	74.2	All	N	Y	Y	Y	Y
23	378	Parallel	50.5	70+	75.0	≥200IU	N	Y	Y	Y	Y
24	489	Factorial	100.0	65-77	71.0	None	N	N	Y	Y	Y
25	160	Parallel	100.0	50-65	59.1	All	N	Y	Y	Y	Y
26	305	Parallel	100.0	60-70	63.8	All	N	N	Y	Y	Y

27	107	Parallel	57.9	60+	81.0	≥1000 IU	Y	N	Y	Y	Y
28	68	Parallel	72.1	65+	77.9	Any D2 or ≥1000 IU/day D3	N	N	Y	Y	N
29	273	Parallel	100.0	57-90	66.4	All	N	N	Y	Y	Y
30	5110	Parallel	41.9	50-84	65.9	≥600 IU if 50-70 years old; ≥800 IU if 71-84 years old	N	N	Y	Y	Y
	Mean: 1371.4 Median: 336.5 IQR: 170-1863.5	86.7% were parallel designs	Mean: 80.3 Median: 85.0 IQR: 61-100			30.0% had no exclusion of Vitamin D supplementation	30.0% recruited participants from a facilitated/long term care facility	30.0% recruited participants based on falls history		76.7% reported double-blinding	

Y – Yes; N – No; NR – Not reported

Supplemental Table S3: Vitamin D assessment at baseline and follow-up

Trial	Serum 25(OH)D Assessment			
	Type of Assay	Eligibility Criteria	Baseline, mean [median] ng/mL	Assessment at Follow-up
1	NA	NA	NA	N
2	RIA	≤ 50nmol/L = 20ng/mL	10.0*	Y
3	RIA	NC	[12]	Y
4	NR	NC	[17]	Y
5	NR	NC	NA	Y
6	RIA	≤ 12ug/L = 12ng/mL	10.4	Y
7	RIA	NC	11.7	Y
8	RIA	25-90nmol/L = 10-36ng/mL	89% < 24ng/mL[†]	N
9	HPLC	NC	15.2[‡]	Y
10	NR	NC	NA	N
11	PBA	NC	29.5	Y
12	NR	NC	19.5	Y
13	RIA	NC	23.2	Y
14	RIA	NC	22.6[‡]	Y
15	RIA	≤ 24.0ng/mL	17.9	Y
16	RIA	≤ 78nmol/L = 31.3ng/mL	21.8	Y
17	RIA	NC	19.9[‡]	Y
18	RIA	NC	19.6[§]	Y
19	RIA	NC	26.4[‡]	Y
20	ELISA	NC	18.8[‡]	Y
21	LC/MS/MS	NC	19.3	Y
22	IEA	NC	26.9	Y
23	RIA	NC	2.6% < 12ng/mL[†]	Y
24	PBA	NC	31.6	Y

25	HPLC	NC	16.0	Y
26	LC/MS/MS	NC	13.5	Y
27	LC/MS/MS	<40ng/mL	23.0	Y
28	CLIA	NC	20.9	Y
29	RIA	<20ng/mL	14.6	Y
30	LC/MS/MS	NC	25.2	Y
		23.3% of trials had eligibility criteria based on baseline serum 25(OH)D levels.		90.0% assessed 25(OH)D at follow-up

Y – Yes; N- No; NC – Not a criterion; NA - not assessed; nmol/L measurements were converted to 1ng/mL with the conversion factor of 1ng/mL = 2.496nmol/L

Type of Assay: RIA - radioimmunoassay; PBA - protein-binding assay; NR - not reported; HPLC - high performance liquid chromatography; ELISA - enzyme-linked immunosorbent assay; IEA - immunoenzymatic assay; LC/MS/MS - liquid chromatography with tandem mass spectrometry; CLIA - chemiluminescent immunoassay (CLIA)

* reported as 25.1nmol/mL, which is likely an error. Calculated based on 25.1nmol/L

† unclear mean/median

‡ analyzed in subset, reported as ng/mL in primary paper, but should have been nmol/L. Value reflects updated units.

§ weighted average of medians

Supplemental Table S4: Trial intervention and follow-up characteristics

Trial	Comparison Group	Concomitant calcium for comparison group	VitD Intervention	Dose	Concomitant Calcium	Mode of delivery	Frequency of VitD supplementation	Compliance Reporting
1	placebo	None	D3	400 IU	None	po	daily	N
2	placebo	600mg	D3	800 IU	600mg	po	daily	Y
3	placebo	1200mg	D3	800 IU	1200mg	po	daily	N*
4	placebo	None	D2	300,000 IU	None	po	single dose	Y
5	placebo	None	D3	100,000 IU	None	po	every 4 months	N
6	placebo	None	D2	600,000 IU	None	im	single dose	N
7	no placebo	None	D2	300,000 IU	None	im	yearly	N
	no placebo	None	D2	300,000 IU	1000mg	im	yearly	
	no placebo	None	D3	800 IU	1000mg	po	daily	
8	placebo	600mg	D2	10,000 IU	600mg	po	weekly†	Y
9	placebo	None	D3	800 IU	None	po	daily	Y
	placebo	1000mg	D3	800 IU	1000mg	po	daily	
10	no placebo	None	D3	800 IU	1000mg	po	daily	Y
11	placebo	None	D3	700 IU	500mg	po	daily	Y
12	placebo	None	D2	200 IU	None	po	daily	Y
	placebo	None	D2	400 IU	None	po	daily	
	placebo	None	D2	600 IU	None	po	daily	
	placebo	None	D2	800 IU	None	po	daily	
13	placebo	1200mg	D3	800 IU	1200mg	po	daily	Y
14	placebo	None	D2	300,000 IU	None	im	yearly	N
15	placebo	1000mg	D2	1000 IU	1000mg	po	daily	Y
16	placebo	1000mg	D3	800 IU	1000mg	po	daily	Y
17	no placebo	None	D3	800 IU	1000mg	po	daily	Y
18	placebo	None	D3	500,000 IU	None	po	yearly	Y
19	placebo	None	D3	150,000 IU	None	po	every 3 months	Y
20	no placebo	None	D2	2.5mg	None	po	every 3 months	N

21	24,000 IU D3	None	D3	60,000 IU	None	po	monthly	Y
	24,000 IU D3	None	D3 + calcifediol	300,000 IU/ 300 ug	None	po	monthly[‡]	
22	placebo	None	D3	800 IU	None	po	daily	Y
23	placebo	None	alfacalcidol	1ug	None	po	daily	N
24	placebo	None	calcitriol	0.25ug	None	po	2/day	Y
	ERT	None	calcitriol	0.25ug	None	po	2/day	
	HRT	None	calcitriol	0.25ug	None	po	2/day	
25	placebo	None	D3	1000 IU	None	po	daily	Y
26	Placebo	None	D3	400 IU	None	po	daily	Y
	Placebo			1000 IU		po	daily	
27[§]	12000 IU VitD3	None	D3	100,000 IU	None	po	monthly	N
28	400IU Vit E	None	D3	100,000 IU	None	po	monthly	Y
29	Placebo	Up to 1200-1400mg/d	D3	400 IU	Up to 1200-1400mg/d total intake	po	daily	Y
	Placebo		D3	800 IU		po	daily	
	Placebo		D3	1600 IU		po	daily	
	Placebo		D3	2400 IU		po	daily	
	Placebo		D3	3200 IU		po	daily	
	Placebo		D3	4000 IU		po	daily	
	Placebo		D3	4800 IU		po	daily	
30	Placebo	None	D3	100,000 IU	None	po	monthly	Y
					28.3% of intervention arms included concomitant calcium	91.3% of intervention arms used oral formulations of Vit D	67.4% of intervention arms used daily supplementation	

Mode of delivery: po – oral; im- intramuscularly

* supplementation administered by staff to ensure compliance, but rates not explicitly reported

† until Nov 1988, then 1,000 IU daily

‡ 60,000 IU drink monthly + 2x1200 IU D3 pills/month + 300ug calcifediol/month

§ those already taking 400-1000 IU were randomized to 100,000 IU or placebo; those taking less than 400 IU/day were randomized to 100,000 monthly or 12,000 monthly; all continued usual supplementation

Supplemental Table S5: Outcome assessment and reporting characteristics

Trial	Falls as primary outcome	Falls definition reporting	Primary Falls Ascertainment Method	Frequency of Recall	Total Follow up Duration [Median/Mean]	# persons who fell	Average Falls/Group	# persons with recurrent falls	time to first fall
1	N	Y	Diary	at-event	28 weeks	Y	Y	Y	N
2	N	Y	Questionnaire	end of follow up	8-week intervention + 1 year follow up	Y	Y	N	N
3	Y	Y	Nurse-report	at-event	12 weeks	Y	Y	Y	N
4	Y	N	Diary	at-event	6 months	Y	Y	N	Y
5	N	N	Questionnaire	yearly	5 years	Y	N	N	N
6	N	Y	Diary	at-event	6 months	Y	Y	N	N
7	Y	N	Interview	3, 6, and 12 months	1 year	Y	N	N	N
8	Y	Y	Nurse-report	at-event	2 years	Y	Y	N	Y
9	N	N	Questionnaire	every 4 months*	[45 months]	Y	Y	Y	Y
10	N	N	Questionnaire	6 months	[25 months]	Y†	N	N	N
11	N	Y	Postcards	at-event	3 years	Y	Y	Y	N
12	Y	Y	Nurse-report	at-event	5 months	Y	Y	Y	Y
13	Y	Y	Nurse-report	at-event	[30 days]	Y	Y	N	Y
14	N	N	Questionnaire/Interview	every 6 months	3 years	Y	N	N	N
15	Y	Y	Interview	every 6 weeks	1 year	Y	N	Y	N
16	Y	Y	Diary	at-event	1 year treatment + 8 months follow up	Y	Y	N	Y
17	Y	Y	Interview	yearly (4 months for subsample)	3 years	Y	Y	Y	N
18	Y	Y	Postcards	at-event	3-5 year intervention + 1 year follow up	Y	Y	Y	N

19	Y	Y	Diary	at-event	9 months	Y	N	Y	N
20	Y	N	Nurse-report	at-event	[10 months]	Y	Y	N	N
21	N	Y	Diary	at-event	1 year	Y	Y	N	N
22	Y	Y	Diary	at-event	2 years	Y	Y	Y	Y
23	Y	Y	Diary	at-event	36 weeks	Y	Y	N	N
24	N	N	Interview	6 weeks, 3, 6, 12, 18, 24, 36 months	3 years	Y	Y	N	N
25	Y	Y	Interview	end of follow-up	9 months	Y	N	Y	N
26	N	N	Interview	every 2 months	1 year	Y	N	N	N
27	N	N	Chart Review	end of follow-up	12 months	Y	Y	Y	Y
28	Y	N	Calendar	at-event	5 months	Y	Y	N	N
29	N	Y	Interview	every 3 months	12 months	Y	N	N	N
30	N	Y	Questionnaire	monthly, then switched to every 4 months	[3.4 years]	Y	N	Y	Y
	56.7% of studies looked at falls as a primary outcome	63.3% of studies reported definition of falls	53.3% utilized at-event reporting of falls			100.0% compared # fallers	66.7% compared cumulative number of falls	40.0% compared recurrent fallers	30.0% compared time to first fall

Y – Yes; N – No;

* sent questionnaire every 4 months to ask if participant fell in the past week

† only reported OR at 6 months and 12 months
